# Anisotropy of hydrogen diffusion in nickel single crystals: the effects of self-stress and hydrogen concentration on diffusion

**DOI:** 10.1038/srep45041

**Published:** 2017-03-22

**Authors:** J. Li, A. Oudriss, A. Metsue, J. Bouhattate, X. Feaugas

**Affiliations:** 1 LaSIE, Université de la Rochelle, CNRS-UMR 7356, France

## Abstract

Hydrogen diffusion has an important role in solute-dependent hydrogen embrittlement in metals and metallic alloys. In spite of extensive studies, the complexity of hydrogen diffusion in solids remains a phenomenon that needs to be clarified. In this paper, we investigate the anisotropy of hydrogen diffusion in pure nickel single crystals using both an experimental approach and a thermodynamic development. As a first approximation, experimental data from electrochemical permeation and thermal desorption spectroscopy are described using the classical Fick’s laws and an apparent diffusion tensor. Within a thermodynamic framework, the diffusion equation can be derived from Fick’s laws with an apparent diffusion coefficient which contains an added solute content dependent term β. This term is due to the elastic strain field associated with the insertion of solute atoms. For nickel crystals, the dependence of β on the crystallographic orientation arises from the elastic anisotropy. Additionally, our results elucidate the discrepancies between the thermodynamic model and experimental observations of the effect of the solute concentration on the diffusion process. Moreover, this highlights the importance of the impact of hydrogen on vacancy formation and the subsequent consequences on the anisotropy of the apparent diffusion coefficient.

Diffusion in solids is fundamental in the art and science of materials and thus a key point for solid-state physics, physical chemistry and metallurgy. Processes connected with diffusion pose some problems such as creep, corrosion and embrittlement. Hydrogen diffusion is extremely fast in solids, even at low temperature, and can impact the fracture resistance of a material. Consequently, getting a better understanding of hydrogen diffusion in solids is a challenge for scientists and engineers who design and build operating materials.

The diffusion coefficient is generally considered to be constant according to Fick’s laws for a given temperature, despite the fact that hydrogen flux depends on the hydrogen concentration and the chemical potential. Chemical potential in particularly is directly influenced by the applied stresses^1^, and the induced stresses^2^,^3^,^4^,^5^ associated with the incorporation solutes or vacancies. Moreover, many studies have pointed out that diffusion in solids depends on defect density and distribution (point defects, dislocations, grain boundaries, inter-phases boundaries, triple junctions, etc.) but their impact is still controversial. In most cases, the influence of defects on diffusion was considered to be an indirect impact associated with trapping mechanisms^6^,^7^,^8^,^9^,^10^,^11^,^12^. However, defects can also act as high diffusivity paths because the mobility of solutes along such defects is usually much higher than in the lattice^13^,^14^,^15^,^16^,^17^,^18^,^19^. All these effects, (defects, concentration and stress) have a significant influence on the apparent diffusion coefficient in complex metallurgical structures (polycrystals, bicrystals, biphasic structures, precipitation hardening alloy, etc.). On the other hand, vacancy formation (V-H clusters) enhanced by the presence of hydrogen has been observed in many Metal–Hydrogen alloys^20^,^21^,^22^. This phenomenon affects hydrogen solubility and diffusion, although the factors that determine the equilibrium vacancy concentration have not been well identified yet^23^. Consequently, the interaction between defects, internal and applied stresses and hydrogen remain major key points for the diffusion processes.

For single crystal solid phases, the impact of crystallographic orientation on the diffusion coefficient D has not been fully elucidated^13^,^24^,^25^. As reported by Brass *et al*.^25^, the hydrogen diffusion coefficient D in nickel single crystals is different between orientations 〈110〉 and 〈100〉; they obtained values of D_〈110〉_ = 6.2 × 10^−14^ m^2^/s and D_〈100〉_ = 5.6 × 10^−14^ m^2^/s. Cao *et al*.^26^ have reported the hydrogen diffusion in nickel polycrystals and nano-textures, and obtained D_〈111〉_ = 85 × 10^−14^ m^2^/s, D_〈110〉_ = 65 × 10^−14^ m^2^/s and D_〈100〉_ = 34 × 10^−14^ m^2^/s. This anisotropy could be an effect of texture or influenced by the nature of grain boundaries. A similar fundamental finding from an atomic point of view was reported by Dederichs & Schroeder^1^, who claimed that the anisotropy of the saddle-point configuration leads to an anisotropy diffusion in cubic crystals. The variable nature of these results led us to question the different metallurgical features which can induce anisotropic diffusivity of solutes in fcc solids with high crystallographic symmetry.

To address this question, we investigated the key factors that determine the anisotropic diffusion of hydrogen in fcc nickel single crystals. For this purpose, hydrogen diffusion in single crystals with different orientations were studied experimentally to highlight the anisotropic diffusivity. Moreover, in a thermodynamic framework, diffusion equations with a self-stress impact on diffusion flux were established to modify Fick’s laws. The analytical results indicated that diffusion is strongly affected by the hydrogen concentration and vacancy formation in relation to a modification of the chemical potential. In both cases, we should emphasize that the anisotropic diffusivity is a direct consequence of the elastic anisotropy.

## Results

**Figure 1:**
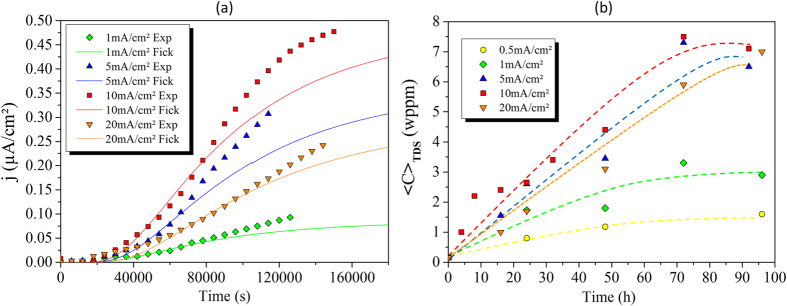
Hydrogen diffusivity and solubility *versus* time. (**a**) Evolution of the current density proportional to the hydrogen flux j as a function of time with different charging conditions (cathodic current densities) for nickel single crystal 〈100〉. Exp: experimental data; Fick: curves calculated using Fick’s law with a constant apparent diffusion coefficient D_app_. (**b**) The average hydrogen concentration 〈C〉_TDS_ in nickel single crystal 〈100〉 as a function of the charging time for different charging current densities.

Hydrogen diffusion in nickel single crystals was studied using electrochemical permeation (EP) and thermal desorption spectroscopy (TDS). The diffusivity of hydrogen through a nickel membrane along the 〈100〉 orientation was measured by EP at 300 K (see the Methods section). The hydrogen diffusion flux *j* as a function of charging time is given in Fig. 1(a). At room temperature, 10 mA/cm^2^ was the most powerful charging condition, which corresponds to the adsorption kinetic regime (the Volmer process).

**Figure 2:**
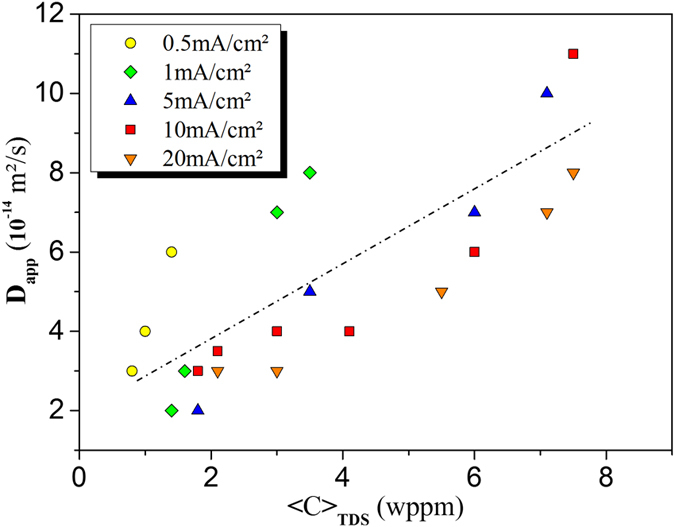
The effect of the average concentration of hydrogen on the apparent diffusion coefficient. The apparent diffusion coefficients were deduced from the simulation of different charging conditions depending on the average concentration of hydrogen in the membrane measured by TDS. For a given charging condition, different 〈C〉_TDS_ correspond to the different charging times (Fig. 1(b)). The simulations were conducted using the FEM code and the classical Fick’s equation where D_app_ was modified each time in order to fit the experimental solubility 〈C〉_TDS_ for several charging conditions.

The EP results showed that hydrogen flux was higher than that calculated by Fick’s laws. The experimental results suggested an acceleration of the diffusion since the diffusion coefficient is considered constant in Fick’s model. However, the quantitative information in Fig. 1(a) on the solubility obtained with the EP tests is not reliable. Indeed, the steady state was never clearly reached. Hydrogen solubility in nickel was measured under various conditions using TDS, and based on the above technique, the results of solubility 〈C〉_TDS_ *versus* charging time are given in Fig. 1(b). Maximum solubility was reached after a critical time and depended on the charging conditions (the hydrogen concentration at the entry side is *C*_0_), in accordance with classical Fick’s law. Further details on the simulations can be found in the Methods section of this paper. More specifically, the change in the apparent diffusion coefficient *versus* the hydrogen concentration, is displayed in Fig. 2, which shows a linear increase in hydrogen diffusivity with hydrogen concentration. A need for a realistic interpretation of the experiments motivated the development of a thermodynamic approach that is introduced in the discussion section.

**Figure 3:**
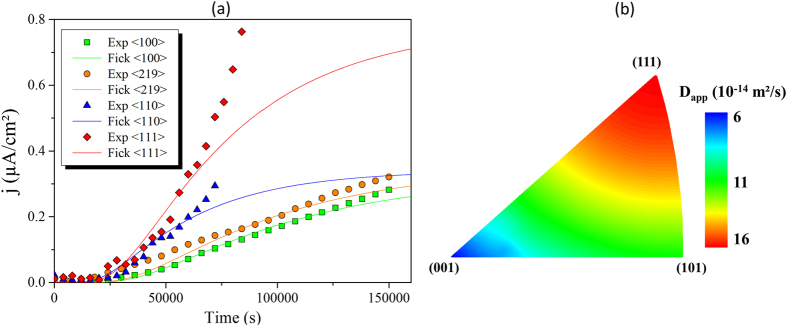
Anisotropic diffusion. (**a**) Hydrogen flux as a function of charging time for different orientations and a charging cathodic current density of 5 mA/cm^2^. Exp〈hkl〉: experimental data; Fick〈hkl〉: curves calculated using Fick’s law with a constant apparent diffusion coefficient D_app_. (**b**) Apparent diffusion coefficients as a function of crystallographic orientations extended from the diffusion tensor (Eq. (1)).

**Figure 4:**
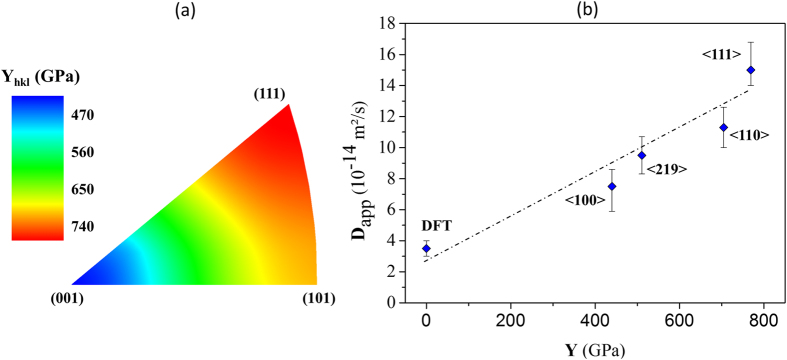
Dependence of the apparent diffusion coefficient *D*_app_ on the anisotropic elastic parameter *Y*. (**a**) Iso-value of *Y*〈hkl〉 in the inverse pole figure. (**b**) The apparent diffusion coefficient *D*_app_ as a function of *Y* for a hydrogen concentration of 7 wppm. The slope of the linear relation between *Y* and *D*_app_ was used to determine the apparent partial molar volume using equation (12).

Based on a number of EP experiments performed at 300 K, hydrogen diffusivity along different crystallographic orientations was determined. A charging current density of 5 mA/cm^2^ was selected for a reliable testing environment to take into account the sample design for the sealing and the experimental containment issues, and in order to respect the Volmer process (a predominance of adsorption kinetics). The hydrogen diffusion flux as a function of charging time is given in Fig. 3(a) for several crystallographic orientations: 〈100〉, 〈110〉, 〈219〉 and 〈111〉. A significant effect of crystallographic orientation on hydrogen diffusivity was observed. Hydrogen diffusion was fastest along the 〈111〉 orientation and slowest along the 〈100〉 orientation. Using Fick’s laws, the apparent diffusion coefficients were determined for each orientation and could be ranked as follow: D_〈111〉_ > D_〈110〉_ > D_〈219〉_ > D_〈100〉_ (Fig. 4(b)). The results obtained were used to determinate an anisotropic diffusion tensor in the cubic coordinate system which respected the symmetries of the fcc structure. Since nickel is fcc structure and possess high degrees of symmetry, the use of a simplification meant that only two index orientations, 〈100〉 and 〈110〉, were necessary to identify the diffusion tensor. Consequently, the 〈219〉 and 〈111〉 orientations were used to validate the approach. The apparent diffusion tensor thus takes then the following form (1) in the crystallographic coordinate system ([100], [010] and [001]):


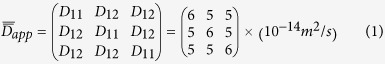


For a more graphic interpretation of our results, the orientation correlation to the diffusion coefficient is represented in the inverse pole figure (Fig. 3(b)). These experimental observations are qualitatively consistent with the data in the literature^25^,^26^ and can be now discussed within the thermodynamic framework.

## Discussion

In the literature, anisotropic diffusion has generally been studied in low symmetry crystals^27^,^28^,^29^, but there remains a controversy concerning more symmetric crystallographic structures^25^,^30^. In this paper, our database demonstrated firstly that hydrogen diffusivity increases with hydrogen content, and secondly that an anisotropy of diffusion is observed in high symmetry fcc nickel single crystals. In the present discussion, we would emphasize that the origin of this behavior lies in the elastic interactions of the solute and the crystallographic structure. Hydrogen incorporation into the interstitial sites of nickel crystals causes an elastic expansion. Thus, a stress is induced by the presence of hydrogen atoms in the elastic solid. Stress, and more generally mechanical fields, are one of the common factors that determine the component of the chemical potential. Therefore, in the present situation, the stress induced or self-stress, resulting from hydrogen incorporation, will affect the transport of the solute in metals as a result of a modification of the stress gradient^2^,^4^,^31^,^32^,^33^. The solution provided by classical Fick’s diffusion equations characterizes hydrogen concentration as a linear variation through the solid at the steady state; we showed here that the stress induced by the incorporation of hydrogen atoms into interstitial sites led to a solution of the diffusion equations that deviated from Fick’s solution. In particular, this stress accelerated the hydrogen diffusion and led to an anisotropic diffusion associated with the anisotropy of elastic coefficients for nickel.

In the context of a phenomenological description of the diffusion, respecting the thermodynamics of irreversible processes, the mobility of the solute is described by assuming a linear relationship between the flux and the driving force. In this case the relationship is expressed by the following equation (2):


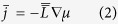



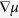
 represents the gradient of the chemical potential for the solute and 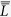 the tensor of transport coefficients^27^,^34^,^35^,^36^. In the particular case of the diffusion of an interstitial element in a crystal lattice, 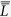 could be expressed in the term of a diffusion tensor 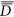 and a concentration of solute *C*_*H*_:


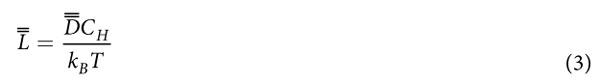


*k*_*B*_ is the Boltzmann constant and *T* the temperature. For the fcc lattice, the symmetries lead to an isotropic diffusion tensor 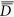 without being affected by the transport tensor 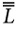. In fact, the gradient of the chemical potential could lead to an anisotropic behaviour in terms of flux. In an isotherm state, the chemical potential^2^,^3^ is considered to be a function of the stress 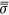 and the hydrogen concentration *C*_*H*_:


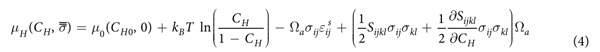


Ω_*a*_ is the atomic volume, *C*_*H*0_ the corresponding standard state concentration, *σ*_*ij*_ the stress tensor, *S*_*ijkl*_ the compliance tensor (the inverse of the stiffness tensor *C*_*ijkl*_) and 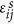 the strain tensor associated with the insertion of a solute in the crystal lattice. The subscripts i, j, k, l = 1, 2, 3, refer to Cartesian coordinates, and repeated indices in the same term imply summation over 1, 2, and 3. In this expression (4), the first term corresponds to the standard state with the absence of stress, the second is the solute distribution entropy, the third term is the elastic strain energy associated with the insertion of a solute, and the fourth term is the mechanical energy input into the system and the solute concentration sufficient to modify the compliance tensor *S*_*ijkl*_. In the absence of external stress, the stress state only depends on the elastic strain from the insertion of a solute in the crystal lattice and leads to modifications of the elastic coefficients. Considering it as a negligible contribution, we get a simplified form of the chemical potential (5):


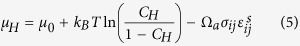


The strain 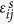, associated with the insertion of a solute (s) in the crystal lattice, is commonly defined as a function of the partial molar volume of hydrogen 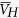 with 
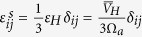
. Depending on the elastic coefficients and the superposition principle, the self-stress *σ*_*ij*_ resulting from the insertion of the solute is obtained from two elastic strain contributions:


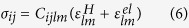


where the strain component 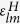 is a hydrostatic strain associated with a variation in the composition and 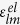 corresponds to a component resulting from stress applied by the crystal lattice on the expansion volume. 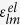 can be deduced from Eshelby’s theory of the inclusion embed in a homogeneous medium with isotropic elasticity coefficients^37^,^38^, and *σ*_*ij*_ can be calculated with (6). (See the supplementary information for a complete development SI-1). Considering the problem of the incorporation of a solute into a crystal lattice can be reduced to a spherical inclusion in a homogeneous medium with anisotropic elastic coefficients, Krivoglaz^39^ proposed an alternative development based on Fourier’s form of the expression of the displacement and the concentration (the supplementary information for the bases of the modeling development is provided in SI-2). Both approaches allow a formulation of the mechanical contribution of the chemical potential: (S1.1, S2.2)


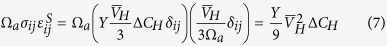



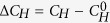
, where 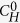 is the initial uniform hydrogen distribution.

For the case of nickel with anisotropic elasticity:


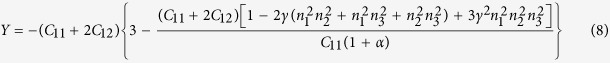


and with isotropic elasticity:


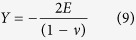


where *C*_*ij*_ are the two standard index components for the stiffness tensor, *E* is Young’s modulus, *ν* is the Poisson’s ratio and *α, γ* are two constants which depend on the elastic constants (see supplementary information SI-2) and 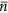 (n_1_, n_2_, n_3_) which is normal to the surface in the crystallographic base of cubic systems. Equations (8) and (9) highlight the fact that *Y* does not depend on the orientation for isotropic elasticity, but it was affected by the orientation of the hydrogen flux for solids which present anisotropic elastic coefficients. The chemical potential introduced in equation (5) now takes the following form:


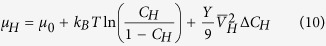


Similar results have been reported by different authors^4^,^31^,^32^,^33^,^40^, mainly in the case of isotropic elasticity. In this development, the partial molar volume 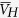 is assumed to be constant.

For a more generic form of the diffusion equation (taking into account anisotropic behavior), the combination of equations (2, 3 and 10) leads to an expression of the hydrogen flux with the apparent diffusion coefficient *D*_*app*_ as a linear relation with *Y*:


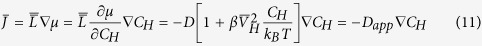


where


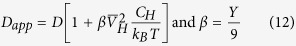


The main conclusion is that the apparent diffusion coefficient, in cubic crystals, depends on the direction of the flux 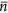 and the hydrogen concentration *C*_*H*_. The evolution of *Y* as a function of crystallographic orientation 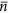
[hkl] is presented in the inverse pole figure describing the fcc structure in Fig. 4(a). The elastic coefficients for nickel^41^, *C*_11_, *C*_12_ and *C*_44_ were 250 GPa, 150 GPa and 120 GPa, respectively. Thus, *Y* is 768 GPa for the 〈111〉 orientation and 440 GPa for the 〈100〉 orientation. This difference has a direct impact on the apparent diffusion coefficient if the partial molar volume and the hydrogen concentration are high enough and at a relatively low temperature. At 300 K, considering equation (11), *D*_app_〈hkl〉 and *Y*〈hkl〉 have a linear relationship (Fig. 4(b)), and the intercept corresponds directly to the diffusion coefficient *D* without the contribution of the self-stress which approximates the results determined by the DFT atomistic calculations: 3 × 10^−14^ m^2^/s by Metsue *et al*.^42^ and 4 × 10^−14^ m^2^/s by Wimmer *et al*.^43^.

According to a compilation of experimental data proposed by Fukai^44^, 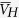 is of the order of 1.8 ± 0.7 × 10^−6^ m^3^/mol, while DFT based on atomistic calculations gave 1.39 × 10^−6^ m^3^/mol in Nazarov *et al*.^45^ and 1.32 × 10^−6^ m^3^/mol in Metsue *et al*.^23^. For a hydrogen concentration of 7 wppm (Fig. 4(b)), we determined that the apparent partial molar volume 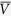 at about 40 × 10^−6^ m^3^/mol inferred from equation (12) is much higher than the theoretical value of about 1.8 ± 0.7 × 10^−6^ m^3^/mol. Thus, the elastic strain associated with the incorporation of hydrogen atoms into the crystal lattice is not sufficient to explain the experimental observations. An alternative explanation to this approach would need to take into consideration the involvement of crystalline defects that may cause stress fields.

The density of dislocations and vacancies is very low at the initial state of nickel single crystals and therefore cannot account for the discrepancies in our results. However, Fukai *et al*.^46^ and more recently Oudriss *et al*.^21^ have pointed out that for nickel, the presence of hydrogen enhances vacancy formation. These observations are supported by the atomistic calculations of Nazarov *et al*.^45^,^47^ and Metsue *et al*.^23^ which highlight the fact that hydrogen decreases the energy of vacancy formation. Thus it seems worthwhile reconsidering the previous approach by taking into account the stress field associated with vacancies, or clusters of vacancies. According to the pioneering work of Eshelby^48^, based on the isotropic elastic continuum approach, the elastic field induced by point defects is expressed as a displacement caused by a center of dilatation corrected by a component termed the constrained displacement field^49^. For a monovacancy or a spherical cluster of vacancies, the isotropic elastic displacement can be used to evaluate a negative dilatation volume 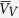. Based on the hypothesis of an additive contribution of the n_v_ vacancies of a cluster, the volume dilatation can be deduced as a function of the expansion volume of a monovacancy. Additionally, atomistic calculations confirm the additive properties of the elastic field of vacancies for bivacancies^50^. Thus if we reconsider the previous approach by taking into account the elastic distortion associated with a cluster of vacancies, the chemical potential introduced in equation (5) takes the following form:


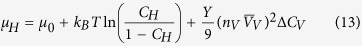


Δ*C*_*V*_ is the excess of vacancies induced by the addition of hydrogen, and the value of the partial molar volume of vacancy^23^ 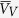 is −2.25 × 10^−6^ m^3^/mol. In accordance with the neglected direct impact of the solute that was highlighted previously, in equation (13) we do not take into consideration the contribution of the term which depends on 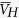.

In quite similar thermodynamic conditions (*j* = 20 mA/cm^2^ and T = 300 K), Oudriss *et al*.^21^ have described a linear relationship between vacancy and hydrogen concentration: Δ*C*_*V*_ = *k*Δ*C*_*H*_ with *k* = 0.15. Equation (13) then takes the form:


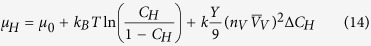


and the apparent diffusion coefficient:


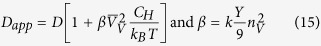


The apparent partial molar volume of 40 × 10^−6^ m^3^/mol derived from equation (12) can now be considered to be 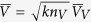 as suggested by equation (15); we deduced that a cluster (radius ≈5 nm) of around forty vacancies is necessary to reach the apparent value.

**Figure 5:**
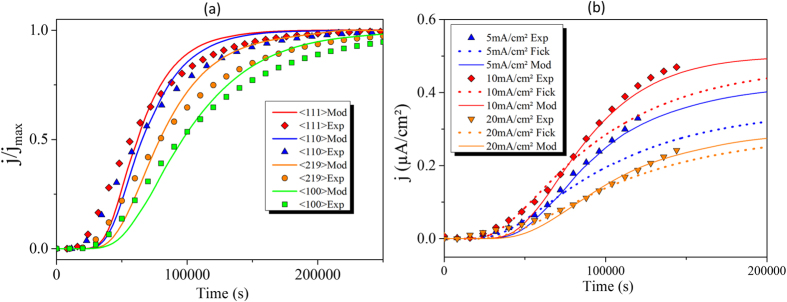
Modeling of hydrogen flux using a modified Fick’s equation. (**a**) Hydrogen flux as a function of the charging time and j_max_ is the hydrogen flux at the steady state, 〈hkl〉 Mod: results calculated for four orientations with Y_〈100〉_ = 440 GPa, Y_〈110〉_ = 705 GPa, Y_〈111〉_ = 768 GPa, and Y_〈219〉_ = 511 GPa; 〈hkl〉 Exp: experimental data with a charging current density of 5 mA/cm^2^. (**b**) The hydrogen flux for the 〈100〉 orientation as a function of diffusion time, Mod: results calculated with Y_〈100〉_ = 440 GPa; Exp: experimental data; Fick: results calculated using Fick’s laws with a constant D_app_.

In order to validate this analysis, we then simulated the electrochemical permeation using a modified Fick’s model based on equations (11 and 15) with FEM (see the Methods section). This step is necessary to take into account the hydrogen concentration gradient across the thick membrane. Figure 5(a) shows the results for different orientations (〈100〉, 〈110〉, 〈111〉 and 〈219〉), thus taking into account the variation of *Y* (hkl) as function of the crystallographic orientation and the expression of β. The calculated curves are in good agreement with the experimental data from the electrochemical permeation tests. Thus, the self-stress effect can explain the diffusion anisotropy by the formation of vacancies during hydrogen diffusion in the nickel sample. Figure 5(b) presents the experimental data, Fick’s diffusion model and a modified Fick’s model based on equation (15) for several charging conditions at the 〈100〉 orientation. The influence of stress on the diffusion is clearly shown since the curves calculated using the modified Fick’s model have a better fit to the experimental curves than the classical Fick’s model.

To conclude, the analysis of coupling stress state/solubility in a thermodynamic framework has shown the importance of the stress state induced by the presence of the solute on the diffusion. Two major effects emerge:

- The apparent diffusion coefficient is a function of hydrogen concentration.

- Self-stress is the origin of the anisotropy of hydrogen diffusion in nickel, which is also a function of the concentration of hydrogen.

In both cases, it is not a direct effect of hydrogen concentration that is highlighted but an induced effect associated with the formation of vacancies during the diffusion of hydrogen. Elastic coefficients were assumed to be constant in this work, which is not the case in reality; however, this can be considered a second order effect. An analysis of influence of hydrogen concentration on the elastic coefficients is in progress^51^.

## Methods

### Electrochemical permeation (EP)

The EP test is the main technique used to detect the mechanisms of diffusion and trapping of hydrogen in different microstructures^21^,^52^. This technique introduced by Devanathan and Stachurski^53^, is composed of two cells separated by a membrane with an exposed surface in contact with an electrolytic solution. The charging side (entry side) of the EP was galvanostatically polarized at a constant cathodic charging current density in 0.1 M NaOH (pH ~ 13), and the current density was predefined according to the cathodic polarization curve. The detection side (exit side) of the EP was maintained with a constant anodic potential of ~630 mV/SSE in 0.1 M NaOH (pH ~ 13), and the hydrogen flux (current density) at the detection side was recorded to study the transport of hydrogen through the membrane. The temperature was maintained at 300 K and both solutions were continuously deaerated by argon gas at 1.4 bar. Before the permeation test, both surfaces of the sample were prepared by mechanical polishing up to grade 4000 SiC; the final thickness of the sample was about 200 ± 20 μm.

### Thermal desorption spectroscopy (TDS)

To quantify the hydrogen concentration and the maximum solubility in nickel, we used Thermal Desorption Spectroscopy (TDS)^21^,^54^. These analyses were performed with a Jobin Yvon Horiba EMGA-621W hydrogen analyzer composed of an impulsion furnace system coupled with a thermal conductivity detector. The procedure used consists involved measuring of the hydrogen concentration in the pre-charged samples by fusion. For this purpose, after hydrogen charging, the specimen (dimension 8 × 4 × 0.3 mm^3^) was introduced into the furnace, where it was instantly heated to 2000 °C and maintained at this temperature for 75 seconds. The desorbed hydrogen was then detected and analyzed by gaseous catharometry. The recorded curve corresponds to the amount of hydrogen detected as function of time. The average concentration of hydrogen in the sample was estimated by measuring the area under the curve. After hydrogen charging, the specimens were mechanically polished with 5 μm SiC grinding paper, then cleaned in acetone before thermal analyses.

### Finite element method (FEM)

Equations (11 and 15) and Fick’s laws were used in Comsol Multiphysics^55^ to describe the variation of hydrogen flux in the membrane for the 〈100〉 orientation with different current densities (0.5, 1, 5, 10 and 20 mA/cm^2^), and different orientations (〈100〉, 〈110〉, 〈111〉 and 〈219〉) with a current density of 5 mA/cm^2^. For reasons of simplification, the model assumed that the hydrogen concentration at the entry side *C*_0_ was constant and the hydrogen concentration at the exit side was null. *C*_0_ was varied each time since the average hydrogen concentration in the membrane could fit experimental data of TDS. The apparent partial molar volume 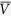 was considered to be constant. Other boundaries were put as no flux, which means the simulation was in one direction only. This approach provided access to the distribution of hydrogen in the membrane and thus allowed us to measure the deviation from the classical Fick’s law, which led to a linear distribution at the steady state.

## Additional Information

**How to cite this article:** Li, J. *et al*. Anisotropy of hydrogen diffusion in nickel single crystals: the effects of self-stress and hydrogen concentration on diffusion. *Sci. Rep.* **7**, 45041; doi: 10.1038/srep45041 (2017).

**Publisher's note:** Springer Nature remains neutral with regard to jurisdictional claims in published maps and institutional affiliations.

## Supplementary Material

Supplementary Information

## References

[b1] Dederichs P. H. & Schroeder K. Anisotropic diffusion in stress fields. Phys. Rev. B. 17, 2524–2536 (1978).

[b2] Larché F. C. & Cahn J. W. The effect of self-stress in solids. Acta metall. 32, 1835–1845 (1982).

[b3] Kirchheim R. & Hirth J. P. Stress and solubility for solutes with asymmetrical distortion fields. Acta metall. 35, 2899–2903 (1987).

[b4] Zhang W. S., Zhang X. W. & Zhang Z. L. Steady concentration distribution of hydrogen in elastic membranes during hydrogen diffusion. J. Alloys Compd. 302, 258–260 (2000).

[b5] Haftbaradaran H., Song J., Curtin W. A. & Gao H. Continuum and atomistic models of strongly coupled diffusion, stress, and solute concentration. J. Power Sources. 196, 361–370 (2011).

[b6] Oriani R. A. The diffusion and trapping of hydrogen in steel. Acta Mater. 18, 147–157 (1970).

[b7] Hirth J. P. Effects of Hydrogen on the properties of iron and steel. Metal Trans A. 11, 861–890 (1980).

[b8] Kirchheim R. Solubility, diffusivity and trapping of hydrogen in dilute alloys. Deformed and amorphous metals-II. Acta Metall. 30, 1069–1078 (1982).

[b9] Cao Y. . Modeling of hydrogen trapping in the deformed Pd and Pd_77_Ag_23_ alloy. Mater. Sci. Eng., A. 379, 173–180 (2004).

[b10] Yao J. & Cahoon J. R. Experimental studies of grain boundary diffusion of hydrogen in metals. Acta Metall. Mater. 39, 119–126 (1991).

[b11] Vlasov N. M. & Fedik I. I. Hydrogen segregation in the area of threefold junctions of grain boundaries. Int. J. Hydrogen Energy. 27, 921–926 (2002).

[b12] Legrand E. . Computational analysis of geometrical factors affecting experimental data extracted from hydrogen permeation tests: III - Comparison with experimental results from the literature. Int. J. Hydrogen Energy. 39, 1145–1155 (2014).

[b13] Brass A. M. & Chanfreau A. Accelerated diffusion of hydrogen along grain boundaries in nickel. Acta Mater. 44, 3823–3831 (1996).

[b14] Yao J. & Cahoon J. R. Theoretical modeling of gain boundary diffusion of hydrogen and its effect on permeation curves. Acta. Metall. Mater. 39, 111–118 (1991).

[b15] Doyle D. M. . The influence of intercrystalline defects on hydrogen activity and transport in nickel. Acta. Metall. Mater. 43, 3027–3033 (1995).

[b16] Arantes D. R. . Hydrogen diffusion and permeation in micro- and nanocrystalline nickel. Acta. Metal. Mater. 41, 3215–3222 (1993).

[b17] Louthan M. R. Jr., Donovan J. A. & Caskey G. R. Jr. Hydrogen diffusion and trapping in nickel. Acta Metall. 23, 745–749 (1975).

[b18] Harris T. M. & Latanision M. Grain boundary diffusion of hydrogen in nickel. Metal.Trans. A. 22, 351–355 (1991).

[b19] Tseng D., Long Q. Y. & Tangri K. Detection of hydrogen permeation on the microscopic scale in nickel. Scripta Metall. 22, 649–652 (1988).

[b20] Fukai Y. . A relation between the vacancy concentration and hydrogen concentration in the Ni–H, Co–H and Pd–H systems. J. Alloys Compd. 404–406, 247–251 (2005).

[b21] Oudriss A. . Grain size and grain-boundary effects on diffusion and trapping of hydrogen in pure nickel. Acta Mater. 60, 6814–6828 (2012).

[b22] Metsue A., Oudriss A., Bouhattate J. & Feaugas X. Contribution of the entropy on the thermodynamic equilibrium of vacancies in nickel. J. Chem. Phys. 140, 104705 (2014). 24628194 10.1063/1.4867543

[b23] Metsue A., Oudriss A. & Feaugas X. Hydrogen solubility and vacancy concentration in nickel single crystals at thermal equilibrium: new insights from statistical mechanics an ab initio calculations. J. Alloys Compd. 656, 555–567 (2016).

[b24] Ebisuzaki Y., Kass W. J. & O’Keeffe M. Diffusion and solubility of hydrogen in single crystals of nickel and nickel-vanadium alloy. J. Chem. Phys. 46, 1378–1381 (1967).

[b25] Brass A. M., Chanfreau A. & Chene J. In Hydrogen Effects on Materials Behavior (ed. Moody N. R. & Thompson A. W.) 19–31 (Minerals, Metals, and Materials Society, Warrendale, PA, 1990).

[b26] Cao Y., Li H. L., Szpunar J. A. & Shmayda W. T. Effects of textures on hydrogen diffusion in nickel. Mater. Sci. Forum, 408–412, 1139–1144 (2002).

[b27] Mehrer H. In Diffusion in solids: Fundamentals, methods, materials, diffusion-controlled processes (ed. Mehrer .) 33–35 (Springer series in solid state science 155, 2007).

[b28] Crank J. In The mathematics of diffusion 5–8 (Clarendon press oxford, 1975).

[b29] Agarwal R. & Trinkle D. R. Light-element in Mg using first-principles calculations: Anisotropy and elastodiffusion. Phys. Rev. B. 94, 054106 (2016).

[b30] Kistner G., Rubin R. & Sosnowska I. Anisotropic diffusion of hydrogen in niobium single crystals. Phys. Rev. Lett. 27, 23 (1971).

[b31] Li J. C. M. & Mehl Medalist R. F. Physical chemistry of some microstructural phenomena. Metal Trans A. 9A, 1353–1380 (1978).

[b32] Larché F. C. Thermodynamics of stressed solids. Solid State Phenomena. 3–4, 205–214 (1988).

[b33] Oriani R. A. The Physical and Metallurgical Aspects of Hydrogen in Metals. ICCF4 (1993).

[b34] Kreuser H. J. In Non-equilibrium thermodynamics and its statistical foundations. 42–44 (Clarendon Press Oxford, 1981).

[b35] Philibert J. In Atom Movements – Diffusion and mass transport in solids. (ed. Philibert J.) 303–309 (Les editions de Physique, Les Uilis, 1991).

[b36] Allnatt A. R. & Lidiard A. B. In Atomic transport in solids. 15–18 (Cambridge university press, 1993).

[b37] Eshelby J. D. The determination of the elastic field of an ellipsoidal inclusion, and related problems. Proc. R. Soc. A. 241, 1226 (1957).

[b38] Mura T. In Micromechanics of defects in solids (ed. Mura T. .) 74–89 (Martinus Nijhoff Publishers, 1987).

[b39] A. Krivoglaz M. In X-Ray and Neutron Diffraction in Nonideal Crystals. (ed. A. Krivoglaz M. .) 95–99 (Springer-Verlag Berlin Heidelberg, 1996).

[b40] Yang F. Q. Interaction between diffusion and chemical stresses. Mater. Sci. Eng., A. 409, 153–159 (2005).

[b41] Ledbetter H. M. & Reed R. P. Elastic properties of metals and alloys, I. Iron, Nickel and Iron-Nickel alloys. J. Phys. Chem. Ref. Data. 2, 3 (1973).

[b42] Metsue A., Oudriss A. & Feaugas X. Hydrogen diffusion in nickel materials. Private Communication. (2017). 10.1038/srep45041 PMC5361197 28327592

[b43] Wimmer E. . Temperature-dependent diffusion coefficients from ab initio computations: Hydrogen in nickel. LM-06K021 (2006).

[b44] Fukai Y. In The metal-hydrogen system basic bulk properties (ed. Fukai Y. .) 104–113 (2005).

[b45] Nazarov R., Hickel T. & Neugebauer J. Ab initio study of H-vacancy interactions in fcc metals: Implications for the formation of superabundant vacancies. Phys. Rev. B. 89, 144108 (2014).

[b46] Fukai Y. . Superabundant vacancy–hydrogen clusters in electrodeposited Ni and Cu. J. Alloys Compd. 356–357, 270–273 (2003).

[b47] Nazarov R., Hickel T. & Neugebauer J. Vacancy formation energies in fcc metals: Influence of exchange-correlation functionals and correction schemes. Phys. Rev. B. 85, 144118 (2012).

[b48] Eshelby J. D. Distortion of a crystal by point imperfections. J. Appl. Phys. 25, 255–261 (1954).

[b49] Mishin Y., Sørensen M. R. & Voter A. F. Calculation of point-defect entropy in metals. Phil. Mag. A. 81, 2591–2612 (2001).

[b50] Yoshioki S. The lattice distortion around the divacancy in cubic metals using the method of lattice statics. J. Phys. F. Metal Phys. 6, 957–963 (1976).

[b51] Hachet G. . Hydrogen consequences on elastic properties of nickel. Private Communication. (2016).

[b52] Frappart S. . Study of the hydrogen diffusion and segregation into FeCMo martensitic HSLA steel using electrochemical permeation test. J. Phys. Chem. Solids. 71, 1467–1479 (2010).

[b53] Devanathan M. A. V. & Stachurski Z. The adsorption and diffusion of electrolytic hydrogen in palladium. Proc. R. Soc. A. 170, 1340 (1962).

[b54] Frappart S. . Hydrogen trapping in martensitic steel investigated using electrochemical permeation and thermal desorption spectroscopy. Scripta Mater. 65, 859–862 (2011).

[b55] Bouhattate J., Legrand E. & Feaugas X. Computational analysis of geometrical factors affecting experimental data extracted from hydrogen permeation tests: I – Consequences of trapping. Int. J. Hydrogen Energy. 36, 12644–12652 (2011).

