# Supplementary material for: Anisotropy of hydrogen diffusion in nickel single crystals: the effects of self-stress and hydrogen concentration on diffusion
Source: Sci Rep. 2017 Mar 22;7:45041. doi: 10.1038/srep45041 (PMC5361197; doi:10.1038/srep45041)
Supplement: Supplementary Information [file srep45041-s1.pdf]

Anisotropy of hydrogen diffusion in nickel single crystals:

the effects of self-stress and hydrogen concentration on diffusion

J. Li*, A. Oudriss, A. Metsue, J. Bouhattate, X. Feaugas*

LaSIE, Université de la Rochelle, CNRS-UMR 7356, France.

J. Li*: jiaqi.li@univ-lr ; X. Feaugas*: xavier.feaugas@univ-lr.fr

Supplementary information

SI-1 Thermodynamic framework: isotropic conditions - The strain of a volume Ω near of an atom of hydrogen $\varepsilon_{ij}^{\Omega}$ is the sum of a component associated to the composition variation ε_{ij}^{H} and a component resulting from the stress applied by the crystal lattice on the volume $\varepsilon_{ij}^{el} = S_{ijkl}\sigma_{kl}^{H}$ (the superposition principle): $\varepsilon_{ij}^{\Omega} = \varepsilon_{ij}^{H} + S_{ijkl}\sigma_{kl}^{H}$. There is a direct relationship between the tensors $\varepsilon_{ij}^{\Omega}$ and ε_{ij}^{H} : $\varepsilon_{ij}^{\Omega} = S_{ijkl}^{I}\varepsilon_{ij}^{H}$, where S_{ijkl}^{I} is the Eshelby's tensor[21, 22]. For the case of an isotropic elastic solid and the description of the solute as a dimensionally stable sphere, we could obtained: $S_{1111}^{I} = S_{2222}^{I} = S_{3333}^{I} = \dfrac{7-5\nu}{15(1-\nu)}$ and $S_{1122}^{I} = S_{2233}^{I} = S_{3311}^{I} = S_{1133}^{I} = S_{2211}^{I} = S_{3322}^{I} = \dfrac{5\nu-1}{15(1-\nu)}$.

The stain is expressed as: $\varepsilon_{11}^{\Omega} = \varepsilon_{22}^{\Omega} = \varepsilon_{33}^{\Omega} = \dfrac{1+\nu}{9(1-\nu)}\varepsilon_H$ with $C_{11kl} = \dfrac{E}{1-2\nu}$ and $\varepsilon_{ij}^{H} = \dfrac{1}{3}\varepsilon_H\delta_{ij}$ E is Young's modulus and ν is Poisson ratio. The stress could then be written as: $\sigma_{11}^{\Omega} = \sigma_{22}^{\Omega} = \sigma_{33}^{\Omega} = \dfrac{-2E}{9(1-\nu)}\varepsilon_H$. The hydrostatic stress takes the form: $\sigma_m^{\Omega} = \dfrac{-2E}{9(1-\nu)}\varepsilon_H$. Without the external stress, we could obtain:

$$\Omega_a\sigma_{ij}\varepsilon_{ij}^{S} = \Omega_a\sigma_m^{\Omega}\delta_{ij}\left(\frac{1}{3}\varepsilon_H\delta_{ij}\right) = \Omega_a\sigma_m^{\Omega}\frac{\overline{V}_H}{\Omega_a} = \frac{-2E}{9(1-\nu)}\varepsilon_H\overline{V}_H = \frac{-2E}{9(1-\nu)}\overline{V}_H^2\Delta C_H \qquad \text{(S1.1)}$$

The chemical potential could be reduced to:

$$\mu_H = \mu_0 + k_B T \ln(\frac{C_H}{1-C_H}) + \frac{2E}{9(1-\nu)}\overline{V}_H^2 \Delta C_H \qquad \text{(S1.2)}$$

SI-2 Thermodynamic framework: anisotropic conditions - Considering now the problem of a solute incorporation in a crystal lattice is reduced to a spherical inclusion in a homogeneous medium with anisotropic elastic coefficients. The strain induced by increasing solute concentration ΔC is expressed as: $\varepsilon_{lm}^{H} = \frac{1}{3}\varepsilon_H \delta_{lm} = L_{lm}\Delta C_H$ with $L_{lm} = \eta\delta_{lm}$ and $\eta = \frac{\overline{V}_H}{3}$. The resulting stress is written as: $\sigma_{ij} = C_{ijlm}(\varepsilon_{lm} - L_{lm}\Delta C_H)$. The equilibrium equation is: $\sigma_{ij} n_j = 0$. Here n_j are the coordinates of the normal to the surface (or the diffusion direction). In order to solve the system of equations and to deduce the stress state as a function of the hydrogen concentration, we assume a linear dependence of the displacement $\overline{u}(\overline{r})$ depending on the concentration ΔC_H, where $\overline{r}$ is the position vector. The Fourier form expressions are given as: $\Delta C_H = C_k \exp(-i\overline{k}.\overline{r})$ and $\overline{u}(\overline{r}) = i\overline{A}_k C_k \exp(-i\overline{k}.\overline{r})$. Where $\overline{k} = k\overline{n}$ and $\overline{A}_k$ is an unknown vector. The strain could be written as:

$$\varepsilon_{lm} = \frac{1}{2}(\frac{\partial u_l}{\partial x_m} + \frac{\partial u_m}{\partial x_l}) = \frac{1}{2}k(n_m A_{kl} + n_l A_{km})C_k \exp(-i\overline{k}.\overline{r}) = \frac{1}{2}k(n_m A_{kl} + n_l A_{km})\Delta C_H$$

Expressions of σ_{ij} and ε_{lm} lead to an expression for the stress tensor as function of the solute concentration: $\sigma_{ij} = C_{ijlm}(\frac{1}{2}k(n_m A_{kl} + n_l A_{km}) - L_{lm})\Delta C_H$. The equilibrium equation is then: $C_{ijlm}(\frac{1}{2}k(n_m A_{kl} + n_l A_{km}) - L_{lm})n_j = 0$. The solution of this equation gives us the expressions of A_i [23]:

$kA_i = \eta X_i, i \in \{1,2,3\}$

Where:

$$X_1 = \frac{1}{1+a}\left(\frac{C_{11}+2C_{12}}{C_{11}}\right)(1-\gamma n_2^2)(1-\gamma n_3^2)n_1$$

$$X_2 = \frac{1}{1+a}\left(\frac{C_{11}+2C_{12}}{C_{11}}\right)(1-\gamma n_1^2)(1-\gamma n_3^2)n_2$$

$$X_3 = \frac{1}{1+a}\left(\frac{C_{11}+2C_{12}}{C_{11}}\right)(1-\gamma n_2^2)(1-\gamma n_1^2)n_3$$

And

$$a = -\gamma\varphi(n_1^2 n_2^2 + n_3^2 n_2^2 + n_1^2 n_3^2) + \gamma^2 \psi n_1^2 n_2^2 n_3^2$$

$$\gamma = \frac{-C_{11}+C_{12}+2C_{44}}{C_{44}}$$

$$\varphi = \frac{C_{11}+C_{12}}{C_{11}}$$

$$\psi = \frac{C_{11}+2C_{12}+C_{44}}{C_{11}}$$

The resulting stress is then deduced: $\sigma_{ij} = C_{ijlm}(\frac{1}{2}(n_m X_l + n_l X_m) - \delta_{lm})\eta\Delta C_H$. The energy associated to this elastic strain is then given:

$$\Omega_a \sigma_{ij}\varepsilon_{ij}^S = \Omega_a (C_{ijlm}(\frac{1}{2}(n_m X_l + n_l X_m) - \delta_{lm})\frac{\overline{V}_H}{3}\Delta C_H)(\frac{\overline{V}_H}{3\Omega_a}\delta_{ij}) = \frac{Y}{9}\overline{V}_H^2 \Delta C_H \qquad \text{(S2.1)}$$

Where, $Y = C_{ijlm}(\frac{1}{2}(n_m X_l + n_l X_m) - \delta_{lm})\delta_{ij}$

For the case of cubic structure, Y has been first published by Larché *et al.*[1] as:

$$Y = -(C_{11}+2C_{12})\left\{3-\frac{(C_{11}+2C_{12})\left[1-2\gamma\left(n_1^2n_2^2+n_1^2n_3^2+n_2^2n_3^2\right)+3\gamma^2n_1^2n_2^2n_3^2\right]}{C_{11}(1+\alpha)}\right\} \qquad \text{(S2.2)}$$

The chemical potential takes the following form:

$$\mu_H = \mu_0 + k_B T \ln\left(\frac{C_H}{1-C_H}\right) + \frac{Y}{9}\overline{V}_H^2 \Delta C_H \, . \qquad \text{(S2.3)}$$
